# Clarifying WHO’s position on the FRAX® tool for fracture prediction

**DOI:** 10.2471/BLT.16.188532

**Published:** 2016-12-01

**Authors:** Nathan Ford, Susan L Norris, Suzanne R Hill

**Affiliations:** a Department of HIV, World Health Organization, avenue Appia 20, 1211 Geneva 27, Switzerland.; b Department of Information, Evidence and Research, World Health Organization, avenue Appia 20, 1211 Geneva 27, Switzerland.; c Department of Essential Medicines and Health Products, World Health Organization, avenue Appia 20, 1211 Geneva 27, Switzerland.

The World Health Organization (WHO) does not currently produce a global estimate of the disease burden attributable to osteoporosis and has long recognized that varying disease definitions and gaps in data complicate the efforts to develop such estimates.^1^

By this statement, the World Health Organization wishes to make clear that the FRAX® tool to evaluate fracture risks of patients is not a “WHO tool” and has not been developed, endorsed, evaluated or validated by WHO, notwithstanding any public statements and claims to that effect.^2^^–^^4^

From 1991 to 2010, the metabolic bone disease unit at the University of Sheffield was designated as a WHO Collaborating Centre, in part to address the abovementioned problem of estimating disease burden. A WHO collaborating centre is an institution designated by the Director-General to carry out activities in support of the Organization's programmes.

While the centre contributed to a 2007 meeting report of WHO’s Scientific Group on the Assessment of Osteoporosis at the Primary Health Level, this report stated only the following in respect of its assessment of the FRAX model: “In Member States without access to densitometry , case-finding strategies can be pursued with use of clinical risk factors alone. The performance characteristics of the FRAX model are at least as good as those provided by peripheral assessment of bone mineral density.”^5^ Neither that report nor the foregoing statement, however, amount to the FRAX model being developed by WHO, nor do they constitute an endorsement by WHO of the FRAX model. 

In 2008, the University of Sheffield released this model as a fracture prediction tool, the FRAX® tool. The subsequent scientific literature and one judicial proceeding reflect controversy over the use of this tool to identify individuals at risk of fracture who may benefit from pharmaceutical treatment to improve bone density.^6^^,^^7^

WHO has no access to the algorithms, coefficients or underlying data on which the FRAX® tool and its national variations have been developed. Consequently WHO is unable to, and does not, express any opinion regarding the scientific value of the FRAX® tool. 

Furthermore, according to our records, WHO has not authorized the use of the WHO name, acronym or emblem in connection with the FRAX® tool, including for the tool’s branding, promotion or sale.  WHO also wishes to clarify that it has not received any income from the sales of subscriptions to the FRAX® tool, whether through the tool’s website or through its iPhone or Android mobile applications.

Lastly, it should be clear that any treatment recommendations^8^ integrated within the FRAX® toolhave not been evaluated by WHO’s Guidelines Review Committee and should not be construed as WHO-endorsed recommendations. Since 2008, recommendations issued by WHO are done so in strict compliance with the process described in the *WHO Handbook for Guideline Development*.^9^ Recommendations are based on systematic reviews of evidence, formal assessments of the certainty of the balance of benefits and harms of an intervention, and explicit consideration of factors such as feasibility, effect on equity, cost and the preferences of persons affected by the recommendations. The guideline development group, which formulates the recommendations, needs to include a broad representation of experts, implementers, and individuals affected by the recommendations, with a clear management plan to minimize potential conflicts of interest.
